# Bioacoustics for species management: two case studies with a Hawaiian forest bird

**DOI:** 10.1002/ece3.1743

**Published:** 2015-10-05

**Authors:** Esther Sebastián‐González, Joshua Pang‐Ching, Jomar M. Barbosa, Patrick Hart

**Affiliations:** ^1^ Department of BiologyUniversity of Hawai'i at Hilo96720HiloHawai'i; ^2^ Department of Global EcologyCarnegie Institution for Science94305StanfordCalifornia

**Keywords:** Algorithm, conservation, Hawai'i ‘Amakihi, song, support vector machine

## Abstract

The management of animal endangered species requires detailed information on their distribution and abundance, which is often hard to obtain. When animals communicate using sounds, one option is to use automatic sound recorders to gather information on the species for long periods of time with low effort. One drawback of this method is that processing all the information manually requires large amounts of time and effort. Our objective was to create a relatively “user‐friendly” (i.e., that does not require big programming skills) automatic detection algorithm to improve our ability to get basic data from sound‐emitting animal species. We illustrate our algorithm by showing two possible applications with the Hawai'i ‘Amakihi, *Hemignathus virens virens*, a forest bird from the island of Hawai'i. We first characterized the ‘Amakihi song using recordings from areas where the species is present in high densities. We used this information to train a classification algorithm, the support vector machine (SVM), in order to identify ‘Amakihi songs from a series of potential songs. We then used our algorithm to detect the species in areas where its presence had not been previously confirmed. We also used the algorithm to compare the relative abundance of the species in different areas where management actions may be applied. The SVM had an accuracy of 86.5% in identifying ‘Amakihi. We confirmed the presence of the ‘Amakihi at the study area using the algorithm. We also found that the relative abundance of ‘Amakihi changes among study areas, and this information can be used to assess where management strategies for the species should be better implemented. Our automatic song detection algorithm is effective, “user‐friendly” and can be very useful for optimizing the management and conservation of those endangered animal species that communicate acoustically.

## Introduction

Populations of many animal species have been and continue to be largely reduced as a consequence of human impact (Dirzo et al. 2014). As a response, large amounts of economic and human resources have been directed to conservation and management programs for threatened and endangered species. However, supporting resources are limited and effective methods that operate in reduced budgets are minimal (Wilson et al. 2006; Sebastián‐González et al. 2011). For example, gathering basic yet valuable information, such as the presence or absence of a species in a specific area, can be time‐consuming, labor intensive, and difficult to implement in remote areas. If the density of the species is low, detecting remaining individuals may require extensive search hours and manpower (e.g., Kovalak et al. 1986; Green and Young 1993), therefore increasing work effort and economic costs. Information regarding the relative abundance of a species in different areas is also valuable for conservation and management programs. This information may be used to assess which areas should be prioritized in conservation plans or to evaluate the temporal trends in the population of a species (Pearce and Ferrier 2001). To acquire this information, managers generally implement traditional point‐count survey methods that require numerous survey hours. Developing alternative, novel techniques to gather information on species presence, distribution, and abundance may reduce input toward costly traditional methods, while increasing the economic resources available for further management strategies.

Many animal species, such as crickets, frogs, whales, and birds, use sounds to communicate, and the presence of these species in a specific place may be confirmed by their acoustic detection (Aide et al. 2013; Marques et al. 2013; Stowell and Plumbley 2014; Heinicke et al. 2015; Merchant et al. 2015). Indeed, bird surveys often rely both in visual and in auditory identification of the target species (Bibby et al. 2000). Moreover, there is not a need for the surveyor to stay for long periods of time in the field, because animal songs and calls can be documented using automatic recorders (Blumstein et al. 2011). These recorders can be left in the field for long periods of time, reducing survey bias related to human presence (Tegeler et al. 2012). However, manually analyzing numerous hours of recordings is also time‐consuming. In recent years, several studies have reported on attempts to automatically identify individual species from many hours of recordings (Bardeli et al. 2010; Briggs et al. 2012; Wellock and Reeke 2012; Keen et al. 2014; Kershembaum et al. 2014). The success of these automatic detectors varies with the method and the targeted species, but most of them are complex algorithms that are difficult to use for managers because they require advanced programming skills (Briggs et al. 2012; Wellock and Reeke 2012; Keen et al. 2014; Kershembaum et al. 2014).

Here, we present a simple algorithm to detect vocalizations of a target species that can be used by ecologists, conservation biologists, and managers without a large background in programming. Recordings from automatic sound recorders may have substantial sound variability depending on community composition, animal behavior, and other environmental factors. This variability needs to be analyzed using statistical approaches that take into account the high‐dimensionality of the data. To account for that, we used a support vector machine, which is a classification method that is able to deal with highly variable data (Camps‐Valls et al. 2004; Mountrakis et al. 2011). We illustrate our detector with two case studies using the Hawai'i ‘Amakihi (*Hemignathus virens virens*). The population of this species, as well as most other native Hawaiian birds, was thought to be restricted to high‐elevation areas where the presence and transmission of introduced avian malaria (*Plasmodium relictum*) is low (Eggert et al. 2008; Hart et al. 2011; LaPointe et al. 2012). However, in recent years, small populations of this species that are apparently resistant to malaria have been detected in low altitude areas, possibly facilitated by an increase in the tolerance of the birds to this disease (Woodworth et al. 2005). One of these areas is a forest adjacent to the Panaewa Zoo on the island of Hawai'i, where some people have heard it, but the presence has not been confirmed. Thus, we recorded environmental sounds at this location and followed our algorithm to confirm the presence of the species in this lowland area. We also used the algorithm to estimate differences in the relative abundance of the ‘Amakihi in two different areas where management strategies for the species may be applied. Finally, we examined the amount of training data required to obtain optimal accuracy in detection. The applicability and cost effectiveness of this method, in a time where resources for conservation and management are limited, renders a broad significance to our study.

## Methods

### Study sites and data gathering

Our study uses information from three areas located in the island of Hawai'i (Appendix S1, Fig. S1). We first collected data from the Pu'u Wa'awa'a Forest Bird Sanctuary (PWW, 05N 0197629E, 2183629N), located at 1200–2000 m of altitude, and the forest near the Panaewa Zoo, Hilo (05N 2175214E, 282474N), located at 20‐50 m of altitude. The Hawai'i ‘Amakihi is abundant in PWW (J. Pang‐Ching, pers. obs.), but its presence is not confirmed in Panaewa. We used the recordings from PWW to train the detection algorithm, and we looked for the ‘Amakihi in Panaewa. The third area is the Hakalau Forest National Wildlife Refuge (05N 0256838E, 2194189N), where ‘Amakihi is present, but at different densities across the refuge. We therefore used data from this site to estimate differences in the relative abundance of the ‘Amakihi in two different areas within the refuge.

In each of these study sites, we collected acoustic information using automatic recorders (Songmeter SM2; Wildlife Acoustics Inc., Concord, MA, Fig. 2E). The recorders were located between 1.5 and 2 m from the ground and separated by at least 150 m. In Panaewa, the Songmeter recorded for 4 min and paused for 2 min (6 min cycles) 60 times from 5:00 am till 11:00 am between the 15 and 31 July 2013, totaling 1680 recording minutes. In PWW, the Songmeters recorded for 5 min and paused for 5 from 6:00 am to 11:00 am between the 10 April and the 9 May 2013. From these recordings, we randomly selected 60 five‐minute files to train the detection algorithm, totaling 300 min of recording. In Hakalau, we recorded at four areas where we expect relative abundances to be different. Within each area, we sampled three different points. We programmed the Songmeter in Hakalau to record from 5:00 am till 8:00 pm recording for 4 min and pausing for 2 min between the 20 June and the 14 July 2013, totaling 37,008 min of recording. Recordings were made in .wav file format at a sampling rate of 44.1 kHz using a single omnidirectional microphone (SMX‐II: Wildlife Acoustics) with a sensitivity of −35 dBV/pa and frequency response of 20 Hz–20,000 Hz.

### Automatic detection algorithm

**Figure 1:**
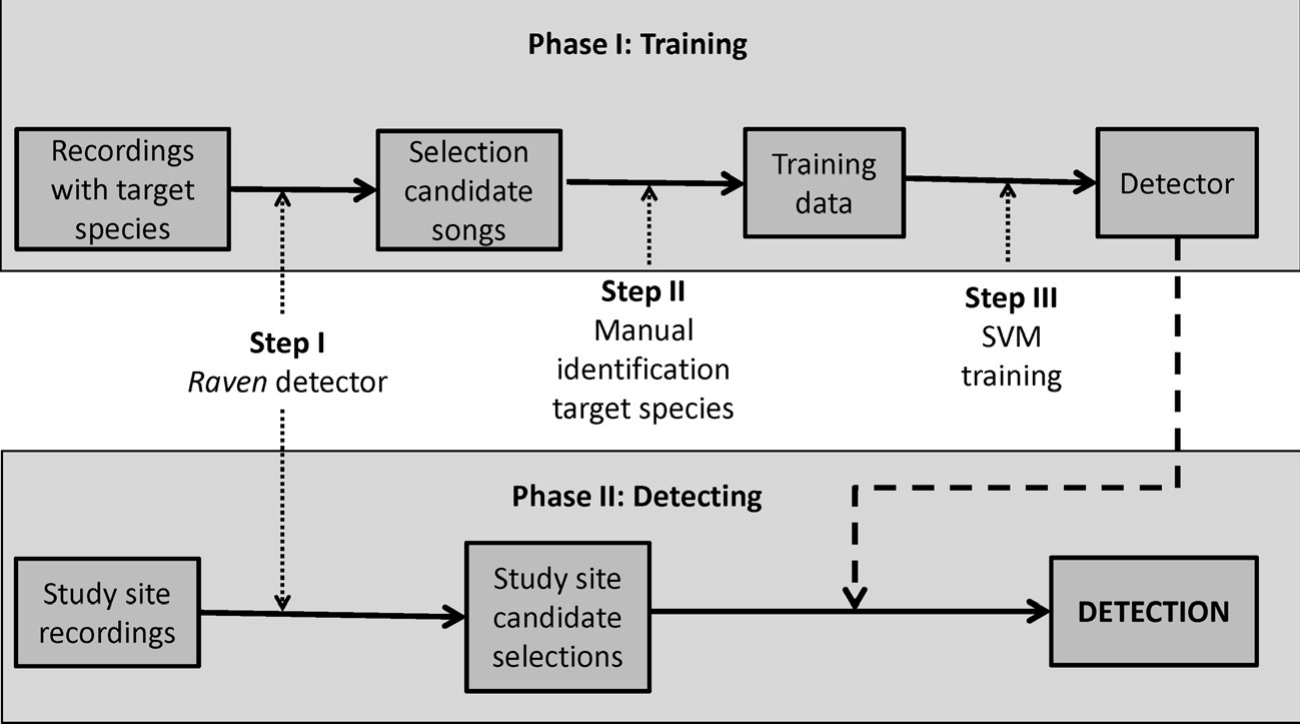
Methodological steps to detect sound‐emitting animal species using *Raven* software and support vector machines (SVM). It consists in two phases: In the Training phase, we first use recordings from an area where the target species is present. We divide the recordings into small candidate songs using the band‐limited energy detector from the *Raven* software (step I), we manually identify which of those songs are from our target species (step II), and we use this information to train the SVM, which is a classification algorithm (step III). In the testing phase, we repeat step I with the recordings from the area of interest, and finally, we use the created detector to identify songs from the target species.

We divided our detection algorithm in two separate phases: Training and Testing. In the Training phase, we used known ‘Amakihi songs to train a data classification tool that is used in the testing phase to detect or count the target species (see Fig. 1). Each of the phases is formed by different steps. The testing phase I starts with the identification of candidate songs that are within the time length and frequency of those of the target species. The second step is the manual identification of those songs that are actually the target. In the third step of the training phase, the manually classified songs are used to train a classification tool (i.e., the support vector machine) that will separate target songs from those from other species or from noise. In the testing phase, we used the detector created in the training phase to detect those songs that are actually from the target species. Each step is described below.

### Training phase, Step I: Interactive detector

As the Songmeters record any sound while they are active, they may record sounds from many natural‐ and human‐originated sources. Thus, we started selecting small candidate sounds that were within the frequency and time ranges of ‘Amakihi songs. To do so, we used the band‐limited energy detector (Mills 2000) from the Raven 1.5 software (Bioacoustics Research Program 2014). This detector estimates the background noise of a sound file and uses this to find sections of signal that exceed a signal‐to‐noise ratio threshold during a specific time and within a specific frequency band. To find the parameters that better described the ‘Amakihi songs, we first manually measured the temporal duration (in seconds) and the maximum and minimum frequencies (in Hz) of 20 ‘Amakihi songs from the PWW recordings and we looked for the configuration of noise parameters that maximized the selection of ‘Amakihi songs from the recordings. ‘Amakihi songs had a duration of between 0.5 and 5 sec, a maximum frequency of 1000 Hz and a minimum frequency of 6100 Hz. Then, we used this configuration to look for candidate ‘Amakihi songs in the 60 PWW training files. For each detected candidate song, we used Raven software to calculate the set of parameters (see Appendix S1, Table S1 for the list of the parameters used) aimed to describe the song.

### Training phase, Step II: Manual song classification

**Figure 2:**
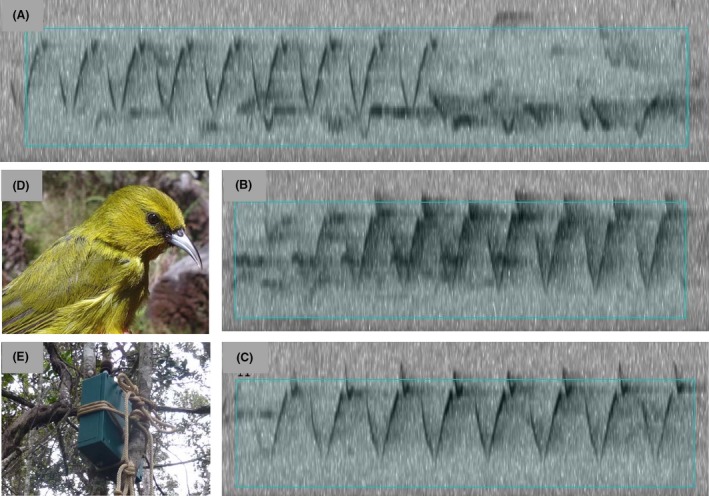
Song classification types and pictures: (A) Bad selection, type 1: song poorly selected/very overlapped with other sounds; (B) medium selection, type 2: ‘Amakihi song not too precisely selected/some overlap; (C) good selection, type 3: song well selected/no overlap, class 3; (D) picture of the Hawai'i ‘Amakihi; (E) picture of a songmeter tied to a tree.

For each of the candidate selections from step I, Raven created a table that can be opened and visualized over the sound spectrogram. We manually opened and classified each of these selections in four classes: 0 (no ‘Amakihi song), 1 (bad selection, ‘Amakihi song poorly selected/very overlapped with other sounds), 2 (medium selection, ‘Amakihi song not too precisely selected/some overlap), and 3 (good selection, ‘Amakihi song well selected/no overlap). See Figure 2A–C for samples of songs 1–3. The songs belonging to class 0 were considered as ‘Amakihi absence, while the songs from classes 1 to 3 belong to ‘Amakihi presence. All the candidate songs from all the classes were used in the analyses. Amakihi songs are very characteristic and it was easy to differentiate them from other species' songs, so the error in the manual identification of the songs was very low.

### Training phase, Step III: Classification using SVM

The support vector machine (SVM, Cortes and Vapnik 1995) is a nonparametric classification tool that uses a multidimensional space to separate elements into binary classes. SVM have already been identified as more accurate than other methods such as Gaussian mixture models for the classification of sound files (Heinicke et al. 2015). In our case, we classified all the candidate songs obtained from step I into presence/absence of ‘Amakihi using a binary SVM model. We first found the best parameters (gamma and cost) in an optimization process using the radial basis kernel function for a large range of values (10^−10^ to 10^10^). We used all the data available for the calculation of these parameters. This procedure ensured the selection of the best parameters, considering trade‐offs between model complexity, overfitting, or underfitting, and number of training data (Ben‐Hur and Weston 2010).

We also calculated the accuracy of our detector in classifying the songs. To do so, we used a cross‐validation approach. We randomly separated 70% of the data as a training set and 30% as validation set. Training files were not used for the validation and validation files were also never used in the training analysis. The training set was used to predict binary classes, and the validation set was used to evaluate the model performance. This performance was calculated using the balanced accuracy metric (Féret and Asner 2012):

$$\text{BAC}=\frac{\text{P(A)}+\text{P(B)}}{2}\times 100$$

where BAC is the balanced accuracy of the SVM predictions to separate two classes, P(A) is the proportion of songs correctly classified as ‘Amakihi presence, and P(B) is the proportion of songs correctly classified as ‘Amakihi absence. We repeated this procedure 1000 times by random selection of the training and validation of dataset, and subsequently, we calculated the mean and standard deviation (SD) of BAC. We also included the area under the ROC curve as a measure of accuracy. This curve represents the relationship between the true‐positive rate and the true negative rate at different thresholds and ranges from 0.5 (low accuracy) to 1 (high accuracy). All the analyses were performed using R 2.15.1 (R Development Core Team 2012) with the ROCR (Sing et al. 2005) package.

We include the R code to run the SVM and calculate the BAC in Appendix S3 and the details on how to get the dataset in the format required for the SVM in Appendix S2.

Finally, we also wanted to test the effect of the amount of data used to train the SVM. To do so, we recalculated the BAC using random subsets of the data (90, 80, 70, 60, 50, 40, 30, 20, and 10%). We repeated the process 100 times and calculated the mean and the SD of the BAC for each proportion of the data.

### Testing phase: detection

The testing phase starts with the same step I as the training phase, using the limited energy detector to identify candidate songs from the target species. We looked for songs from all the recordings at the study areas (in this case, from Panaewa and Hakalau), which are where we aim to detect presence and measure the relative abundance of ‘Amakihi. Then, we used the best SVM model (higher BAC) to automatically identify (1) whether any of the candidate songs from Panaewa was classified by the SVM as ‘Amakihi and (2) the number of candidate selections from Hakalau that were classified as ‘Amakihi.

### Relative abundance analyses

We calculated the relative abundance of the ‘Amakihi by counting the number of detections from the SVM per minute (i.e., total number of detections in a recording file/number of minutes recorded in that file). As the recordings were taken at the same time, the number of vocalizations detected may be a function of the number of individuals at each area and can be used to compare relative abundance among areas. Then, we tested the performance of our relative abundance estimation comparing our results with an ‘Amakihi density estimation calculated at the same area in April–May 2012 using point counts by Camp et al. (in press). In their article, Camp et al. (in press) estimated ‘Amakihi density (number of birds per hectare) for 265 locations distributed in the same area. We created a 500‐m buffer around each of our study points and averaged the ‘Amakihi density estimated by Camp et al. (in press) for all the locations that were inside the buffer.

We used linear mixed models (LMMs) in R to compare the calls per minute and the density estimation from Camp et al. (in press) among the two study sites. As the data were taken from two different points within each study site, we used point as a random variable in our models. We used the lmer function from the lme4 package (Bates et al. 2014). We constructed two models, one model including only the random variable (point) and a second one including also a variable for the study site as a categorical variable. We compared the models using the Akaike information criterion, which selects the models that show the best trade‐off between likelihood and number of parameters (i.e., the models with the highest explanatory power but the lowest number of parameters). We considered two models that differed in AIC by less that 2 to be equally probable.

## Results

### Detection algorithm accuracy

The interactive detector (band‐limited energy detector) identified 4070 candidate ‘Amakihi songs in the PWW recordings. From those, 1285 were manually identified as ‘Amakihi songs. The detector failed to identify 98 extra ‘Amakihi songs from the recordings (7.0% of the ‘Amakihi songs), but most of them were songs from long distances and were not well captured by the recorder. From this manual selection, 49.6% were from class 1 (very bad selections), 33.6% from class 2 (not good selections) and 16.8% from class 3 (good selections).

**Figure 3:**
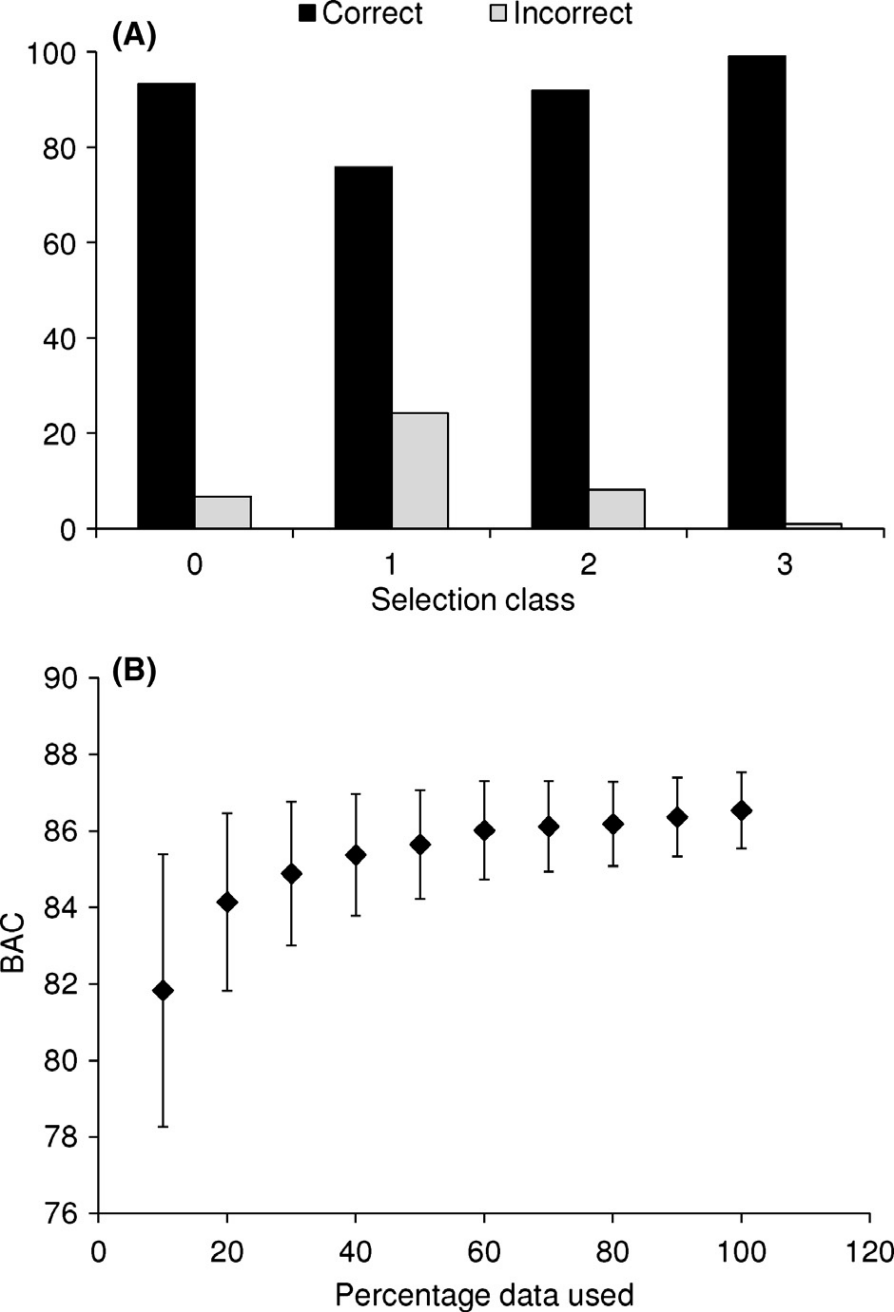
Performance of the support vector machine (SVM) to classify candidate ‘Amakii songs. (A) Proportion of selections that were correctly/incorrectly classified by the SVM according to their classification class. Classification classes as in Figure 2. (B) Average balanced accuracy (BAC) of the SVM using different proportions of the training data. The bars represent the SD of the average BAC. The data were obtained by randomly selecting a proportion of the training data (4070 song selections) and recalculating the BAC 100 times.

The SVM produced a BAC of 86.5% (SD = 1.01) and an AUC of 0.942. Thus, it was highly successful in identifying ‘Amakihi songs among the candidate selections. It detected most of the songs from class 3, while the highest error was in class 1, where the songs were mixed with other noises and the selections often included several other species (Fig. 3A). The number of selections used to train the algorithm slightly changed the BAC, indicating that the algorithm is sensitive to the availability of data (Fig. 3B). However, with only 50% of the original data (2035 selections, including 642 actual ‘Amakihi songs), the BAC was reduced by less than 1%.

### Case studies

We first wanted to confirm the presence of the ‘Amakihi in the lowland habitat near Panaewa Zoo. Our algorithm identified 68 selections as ‘Amakihi songs from 12,617 candidate selections. From those 68 selections, only six were confirmed as ‘Amakihi by a manual inspection of the spectrograms and the songs. Moreover, a manual inspection of 10% of the recordings found 22 additional ‘Amakihi songs that were not detected by the algorithm. The error was higher than in the tests because in the Panaewa recordings, there is a constant cricket sound at a frequency that overlaps with the level at which ‘Amakihi sings, making identification more challenging (see a spectrogram in Appendix S1, Fig. S2). However, the automatic detector allowed us to confirm the presence of the species in the area of interest.

**Figure 4:**
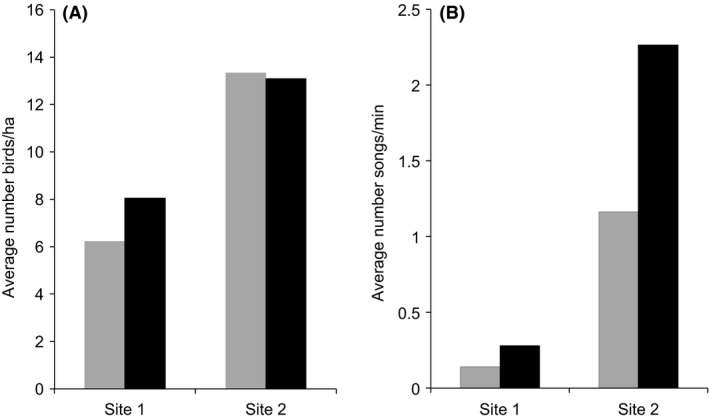
Comparison of ‘Amakihi density and relative abundance among the two study sites. Columns with different colors represent different data collection points within each study site. Both number of birds per hectare and number of songs per minute where significantly higher in site 2 in relation to site 1 (see text for details). (A) Average number of ‘Amakihi per hectare estimated using point counts by Camp et al. (in press). (B) Average number of ‘Amakihi songs per minute at each of the recording sites. The differences in the two replicates in the data for Site 2 from the automatic detector may be caused by differences in habitat within this area. The data from Camp et al. (in press) does not reflect these differences because the points are not located exactly in the same point.

Then, we investigated possible differences in the relative abundance of ‘Amakihi at the two different study areas at Hakalau. The detector found a total of 9252 ‘Amakihi songs from 207,800 candidate selections. The number of calls per minute differed among areas (GLMM, ΔAIC with null model = 437, *P* < 0.001, Fig. 4A), being higher at site 2 than at site 1. These results were in agreement with the density estimation at the same sites by Camp et al. (in press) that also identified a higher ‘Amakihi density at site 2 than at site 1 using the point‐count method (GLMM, ΔAIC with null model = 24.3, *P* < 0.001, Fig. 4B).

## Discussion

In recent decades, researchers and land managers have gone to considerable effort trying to maximize efficiency in the use of the limited resources available for conservation and management. Most efforts are focused on determining optimal locations to establish natural reserves (Arponen et al. 2012; Meller et al. 2014) or assessing which management strategies are most efficient (Wilson et al. 2006; Wintle et al. 2010). However, optimizing survey and monitoring methods has received less attention, while managers continue to implement traditional, cost inefficient methods. In this study, we show how the combination of the band‐limited energy detector (Raven software) with the SVM facilitates the use of song automatic recorders to obtain information on species presence, distribution, and relative abundance, which are central to the effective preparation and implementation of conservation and management plans.

Our algorithm satisfactorily accomplished the objectives of this study, despite not acquiring 100% detection accuracy. First of all, we were able to confirm the presence of the ‘Amakihi in the lowland wet forests of Panaewa. This provides further evidence that the ‘Amakihi may be recolonizing lowland habitats on Hawaii Island as they gain resistance to avian malaria (Spiegel et al. 2006). Our results hold value for conservation strategies as a baseline driving force for management, simply understanding the presence or absence of species in a given area (Gómez de Silva and Medellin 2001). As discussed, simple information on species distribution and abundance may be costly to acquire. However, using automatic sound recorders, coupled with proper computer algorithms, is a cost‐effective alternative to detect the presence/absence of acoustic species. Furthermore, species detection algorithms created using our approach may also be used to accurately identify the distribution of many animal species, by strategically recording near the apparent limits of the distribution of the species. Detailed knowledge on species distribution within a given area may be very useful to assess possible effects of human impacts (e.g., construction of infrastructure), management strategies (e.g., elimination of an invasive species), or climate change on the viability of a species (Davies et al. 2006; Porfirio et al. 2014).

In our second study case, we were able to compare the relative abundance of the ‘Amakihi in two different areas and to validate our results with independent data on species density. Even if reliable estimates of the abundance of the species cannot be easily obtained using bioacoustics (but see Marques et al. 2013), many studies have underlined the importance of including at least some qualitative measures of abundance or density in ecology and conservation (Balmer 2002). The estimates of relative abundance obtained using our algorithm can be used to study spatial patterns, such as the effect of habitat fragmentation (dos Anjos et al. 2011), or temporal patterns (i.e., effects of management actions over time, McShea and Rappole 2000).

The automatic identification of species from sound files presents several benefits compared to more classical survey methods such as point counts or transects. First, it reduces the resources required to sample an area for long periods of time because recorders can be left in the field and programmed to record a desired amount of time per day automatically. Also, it does not require bringing experienced surveyors to the field, which may be very important for the identification of some species (Jiguet 2009). These surveyors may be difficult to find and often not available for long periods of time. Second, the invasiveness of the method is very low. Human presence during the surveys may change the behavior of the surveyed animals (Bye et al. 2001), resulting in a detection bias. The recorders are inconspicuous and do not alter the behavior of the animals in the field because no human presence is required during data acquisition. Moreover, the programming skills required to follow the algorithm are not too demanding in comparison with previous described algorithms (Briggs et al. 2012; Wellock and Reeke 2012; Keen et al. 2014; Kershembaum et al. 2014). This facilitates the use of this tool for managers and conservation technicians.

Besides the clear benefits of our algorithm, it also presents several limitations. First of all, it needs recordings from the target species to be able to train the SVM classifier. This can especially be a problem when the target species is very rare in all locations where it exists, and recording enough songs to train the SVM may be more difficult and resource consuming than the common surveying techniques. An additional limitation for those species with complex songs (i.e., formed by several notes which are very different from each other) is to be able to include all the possible song types in the training data so that the SVM can successfully classify any of them. Besides, there may be varying levels of error associated with each step of the algorithm. The band‐limited energy detector in the Raven software may fail to select a song of the target species, especially if the source of the sound is far away from the recorder, or if several sounds are recorded at the same time (e.g., two or more bird species are singing at the same time or a helicopter is flying close to the recorder). However, we detected that this error is very small (<8%), and even if the detector does not capture one of the vocalizations of an individual, birds often vocalize more than one time, increasing the probability that at least some of the vocalizations are captured. The second source of error is the SVM, which presented a BAC lower than 100%. However, the SVM in general overdetected the presence of ‘Amakihi (it sometimes classified as an ‘Amakihi songs that were not actually from this species). Thus, the probability of a species to be present in the area and not detected by the algorithm is low. At the same time, false‐positive detections of target species can be visually identified using the sound spectrogram. It is also important to be aware that the algorithm error can be higher than the reported here in recordings with some types of noises, such as the presence of cricket sounds in the Panaewa recordings.

Relative abundance estimations have other additional limitations. When comparing the relative abundance of the species among different areas, detection errors should be the same in all the areas, so the relative abundances can be compared among them (but not with abundances from other survey methods). For example, it would not be possible to compare the relative abundance between a site where crickets produce an important noise and a second site without them. It is also important to compare recordings done in the same season, because the song rate of the species may vary seasonally (e.g., Leitner et al. 2001). Song propagation is also dependent on the physical constrains that the environment posses to the songs, and song is propagated further in more open areas than in closed environments (Forrest 1994; Mathevon et al. 1996). Thus, relative abundances estimated in areas where the vegetation is very different may be biased and assign higher abundances at open areas than at those with more close vegetation. Finally, the song recorder may not be sensitive enough or the song classifier can fail to distinguish among species that present very similar songs. In this last case, one possible option is to set the classification in two levels. First, train the SVM and classify songs from both species together from the rest of the songs and then train again the SVM to differentiate among the songs that are similar to each other, because this procedure may reduce the data dimensionality facilitating the creation of a decision boundary between classes.

In this study, we have designed the algorithm to detect only one species. However, multispecies detection may also be possible using a similar approach. Two possible options are either using a SVM that classifies more than one class or training two separate detectors using the same data. While we have demonstrated the use of this algorithm with a bird species, it can potentially also be also used for monitoring of frogs, crickets, whales, or any animal that uses sounds to communicate. More research is needed to identify which alternative for multispecies detection is more efficient, of if any adaptation is required for the use of the algorithm with other animals, but our algorithm is a promising first step for a more automated, low‐resource demanding, and less invasive survey method for species that communicate acoustically.

## Conflict of Interest

None declared.

## Data Accessibility

The training data for the detection of the Hawai'i ‘Amakihi and the R codes for the analyses are available in the Supporting Information.

## Supporting information


**Appendix S1**. Supplementary tables and figures.

**Appendix S2**. Details on getting the data from the interactive detector in Raven to the Support Vector Machine in R.

**Appendix S3**. Zipped file containing the R codes, parameters and the training data for the Hawai'i ‘Amakihi detector.
