# Supplementary material for: Bioacoustics for species management: two case studies with a Hawaiian forest bird
Source: Ecol Evol. 2015 Oct 5;5(20):4696–705. doi: 10.1002/ece3.1743 (PMC4670053; doi:10.1002/ece3.1743)

**Appendix S1. Supplementary tables and figures**

**Bioacoustics for species management: Two case studies with a Hawaiian forest bird**

Esther Sebastián-González, Joshua Pang-Ching, Jomar M. Barbosa and Patrick Hart

**Figure S1.** Map of the Island of Hawai’i and the location of the three areas where we recorded. In Pu‘u Wa‘awa‘a Forest Bird Sanctuary we recorded to get ‘Amakihi song samples. In Panaewa we used the algorithm to detect the presence of the ‘Amakihi. In Hakalau we used the algorithm to compare the relative abundance of the ‘Amakihi in four areas. Map color indicates an elevation gradient from sea level to 4,200 m of altitude.


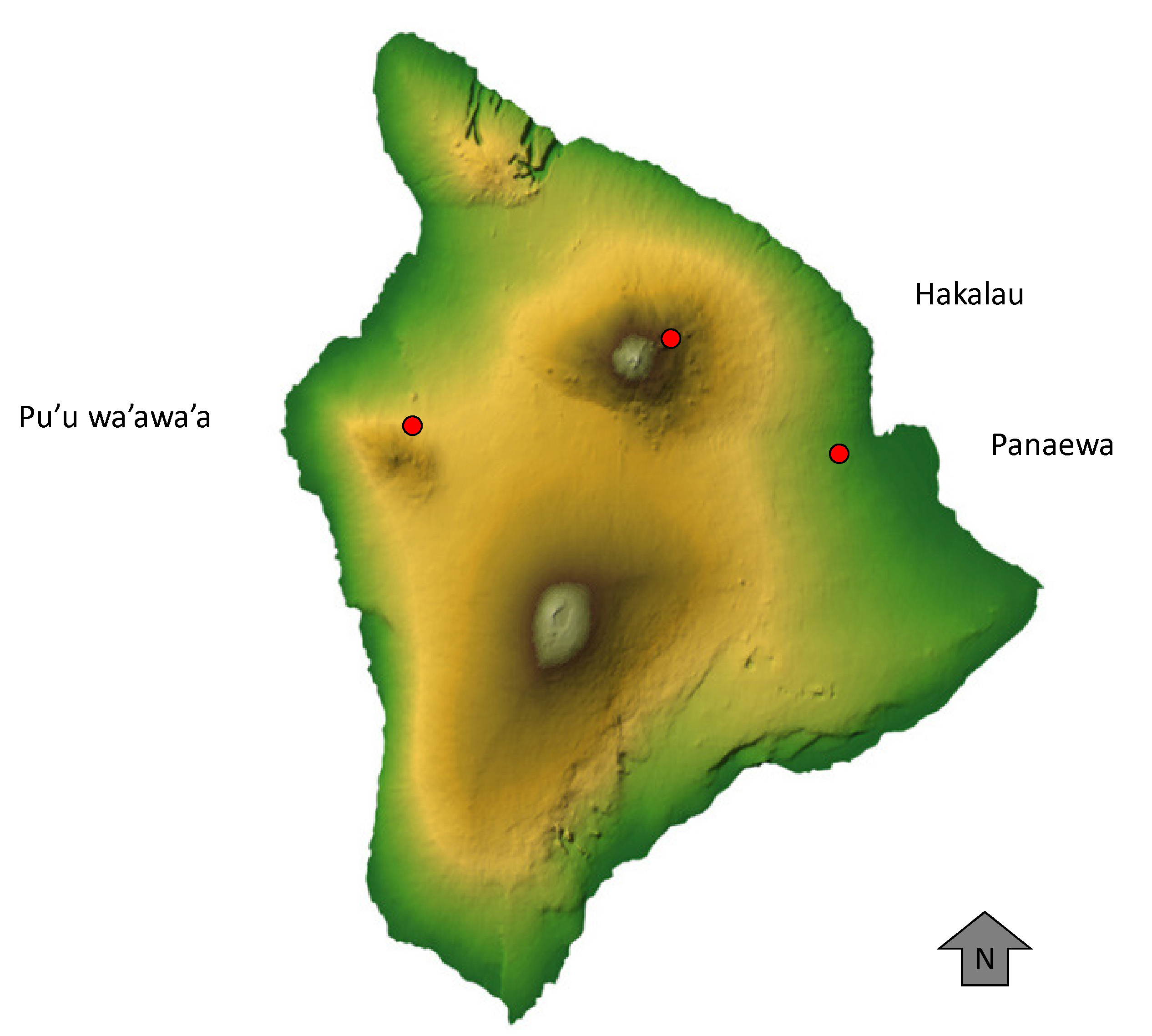


**Table. S1.** List of sound parameters used to detect target bird songs and their units.

Average Amplitude (dimensionless)

Filtered Root-mean-square Amplitude (dimensionless)

Leq (dB)

Maximum Amplitude (dimensionless)

Minumum Amplitude (dimensionless)

Peak Amplitude (dimensionless)

Root-mean-square Amplitude (dimensionless)

SEL (dB)

1^st^ Quartile Frequency (Hz)

3^rd^ Quartile Frequency (Hz)

Aggregate Entropy (bits)

Average Entropy (bits)

Average Power (dB)

Bandwidth 90% (Hz)

Center Frequency (Hz)

Delta Time (s)

Duration 90% (s)

Energy (dB)

Frequency 5 (Hz)

Frequency 95 (Hz)

Inter-quartile Range Bandwidth (Hz)

Inter-quartile Range Duration (s)

Inband Power (dB)

Length (frames)

Maximum Entropy (bits)

Maximum Frequency (Hz)

Maximum Power (dB)

Minumum Entropy (bits)

Peak Frequency (Hz)

Peak Frequency Contour Average Slope (Hz/ms)

Peak Frequency Contour Maximum Frequency (Hz)

Peak Frequency Contour Maximum Slope (Hz/ms)

Peak Frequency Contour Minumum Frequency (Hz)

Peak Frequency Contour Minumum Slope (Hz/ms)

Peak Frequency Contour Number of Inflection Points

Peak Power (dB)

Sample Length (samples)

**Figure S2.** Sonogram of one of the ‘Amakihi songs detected in Panaewa. Note the existence of a dark thin noise between the 4700 and 5700 Hz, which corresponds to crickets’ sounds.


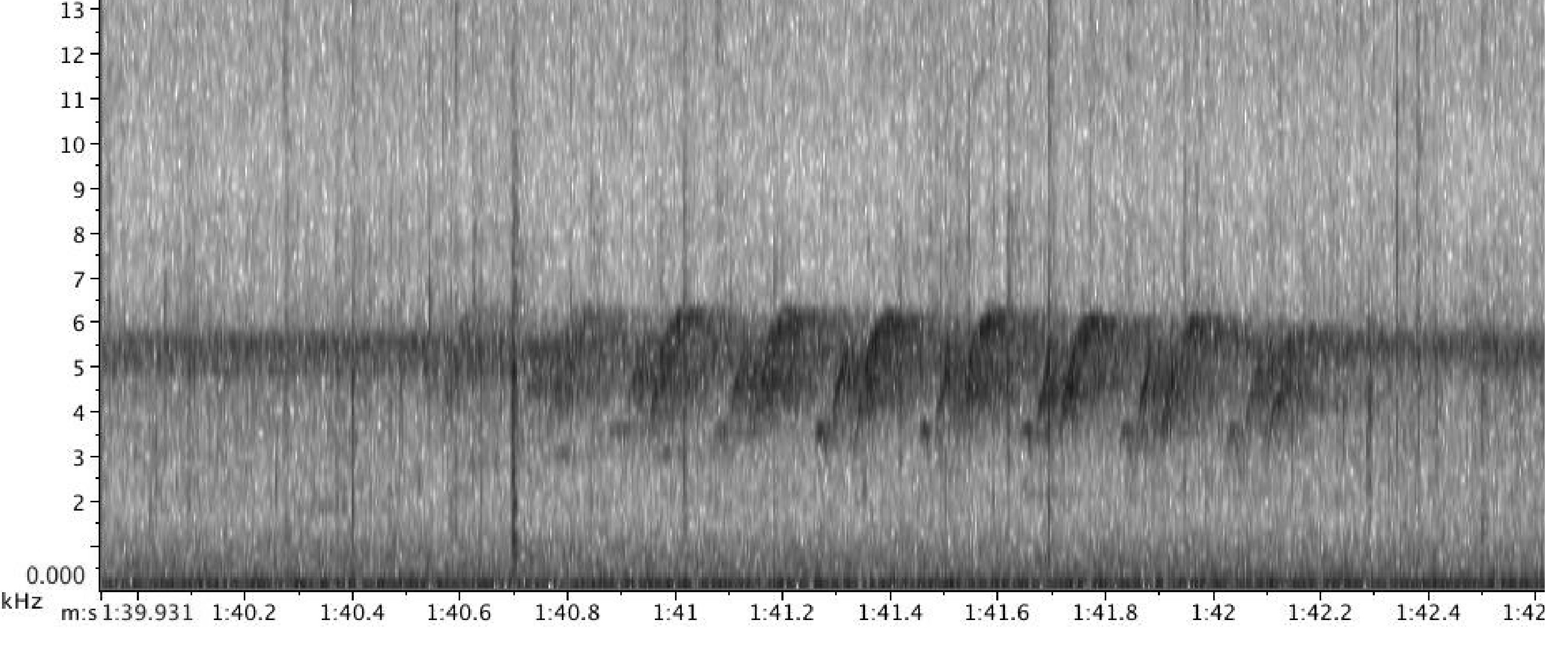

Supplement: Supplementary file 1 — Appendix S1. Supplementary tables and figures. [file ECE3-5-4696-s001.doc]
