# Supplementary material for: Bioacoustics for species management: two case studies with a Hawaiian forest bird
Source: Ecol Evol. 2015 Oct 5;5(20):4696–705. doi: 10.1002/ece3.1743 (PMC4670053; doi:10.1002/ece3.1743)
Supplement: Supplementary file 2 — Appendix S2. Details on getting the data from the interactive detector in Raven to the Support Vector Machine in R. [file ECE3-5-4696-s002.doc]

**Appendix S2. Details on obtaining data from the interactive detector in *Raven* to be used on the Support Vector Machine in R**

**Bioacoustics for species management: Two case studies with a Hawaiian forest bird**

Esther Sebastián-González, Joshua Pang-Ching, Jomar M. Barbosa and Patrick Hart

**Step I**

**1. Band Limited Energy Detector in *Raven***

The first step is getting selections of candidate songs from the recordings. To do so, you should manually select about 20-25 songs from the target species and calculate the maximum and minimum frequency (in Hz) and duration (in s) of all the selections. You may use this information to calibrate the *Target* section of the Band Limited Energy Detector in *Raven* (View-> Interactive detectors -> Band Limited Energy Detector). Then, you should try several combinations of the parameters in the *Noise* calibration trying to maximize the number of selections. It is especially important that the combination of parameters includes all the songs of your target species, even if it also includes songs from other species. In general, low values of *Minimum occupancy* and *SNR threshold* will include a high number of selections, but it is important to try different configurations because the output depends on the recording and on the target species. It may be useful to click on *Enable detector diagnostics* because it explains why the detector did or did not select each part of the spectrogram. It is also worth reading the *Raven* Manual, which includes more details on each of the parameters. Once you found the perfect combination of parameters, save the *Preset* (Preset -> Save), so that you can use it later for all your files.

**2. Configure *Raven* to save the parameters you need**

You also need to configure *Raven* to save all the parameter that will be used afterwards at the SVM. To do so, open one spectrogram and a selection table. Right click on the table and open *Choose measurements*. Include all the measurements that are shown in Table S1, Appendix S1 and save the preset (In the *Choose measurements* window -> Preset -> Save preset).

Now, you need *Raven* to automatically calculate these parameters in any table by default. To do so, go to Edit -> Preferences, and look for the line that says:

# Raven.preset.measurementList.defaultPreset = Default

And change “Default” to the name of the preset you just saved:

Raven.preset.measurementList.defaultPreset = Your.name

Before closing, remove the # symbol in front of the line to make *Raven* read it. Then, close the *Preferences* and close *Raven* so that changes are applied.

**3. Run the detector**

You can now run the detector in several files at the same time. To avoid Raven running out of memory, you may increase the amount of memory allocated to the program. Go to Window -> Memory manager and increase the number for Maximum heap size (We used 12,000).

Then, go to Tools -> Batch detector to run the detector. Select the files you want to analyze (Files window -> Add), indicate the preset you saved before for the Band Limited Energy Detector in the *Configure Detector* window, and indicate the location where you want to save the selection tables with the sound measurements (Table -> Browse).

If you run too many files at the same time, Raven will run out of memory and stop working. The number of files that can be opened at the same time this depends on the computer and on the number of selections that the detector finds. With a Mac OS X (10.9.5) we could run about 250 5-minute long files. This may take quite some time.

**Step II**

**4. Manual song classification**

After going through Step I with your training data (data with the target species), now you need to open the files one by one, create a column called “presence” (right click on the selection table -> Add annotation column) to manually indicate, for each selection, if the target species is present or not.

**Step III**

**5. Joining data to use the SVM**

The data to be used in the SVM should be all in a single file (one file for the Training data, and one file for the Study site recordings). However, we often have several small selection tables if we recorded many short periods of time. Also, the data of the selection tables includes two columns per selection, one for the information on the spectrogram, and one for the information about the waveform), so we need to join the information from the spectrogram and the waveform in one line. To do so, you can use the Merging.files.R code, available on Appendix S3.
